# Leg Extension Power Is a Pre-Disaster Modifiable Risk Factor for Post-Traumatic Stress Disorder among Survivors of the Great East Japan Earthquake: A Retrospective Cohort Study

**DOI:** 10.1371/journal.pone.0096131

**Published:** 2014-04-23

**Authors:** Haruki Momma, Kaijun Niu, Yoritoshi Kobayashi, Cong Huang, Atsushi Otomo, Masahiko Chujo, Hiroko Tadaura, Ryoichi Nagatomi

**Affiliations:** 1 Division of Biomedical Engineering for Health and Welfare, Tohoku University Graduate School of Biomedical Engineering, Sendai, Japan; 2 Department of Epidemiology, School of Public Health, Tianjin Medical University, Tianjin, People’s Republic of China; 3 Department of Medicine and Science in Sports and Exercise, Tohoku University Graduate School of Medicine, Sendai, Japan; 4 School of Nursing in Miyagi University, Sendai, Japan; Univ of Toledo, United States of America

## Abstract

**Background:**

Post-traumatic stress disorder (PTSD) is a common psychological problem following natural disasters. Although pre-disaster risk factors are important for early detection and proactive support, the examination of such has been limited to sociodemographic factors, which were largely unaffected by the disasters. We examined the association between pre-disaster physical functioning and lifestyle and PTSD symptoms five months after the earthquake in the Great East Japan Earthquake survivors who were participating in a pre-existing cohort study.

**Methods:**

We designed a retrospective cohort study of a cooperative association in Sendai from August 2010 to August 2011. In 2010, lifestyle, physical condition, and sociodemographic factors were examined by self-reported questionnaires completed by 522 employees of this organization. We also measured the leg extension power of all the participants. PTSD symptoms were evaluated by the Japanese version of the Impact of Event Scale-Revised (IES-R-J) following the earthquake of 2011.

**Results:**

In multivariate linear regression analysis, leg extension power (*β* = –0.128, *P* = 0.025), daily drinking (*β*  = 0.203, *P* = 0.006), and depressive symptoms (*β*  = 0.139, *P* = 0.008) were associated with total score of the IES-R-J among men. Moreover, for the IES-R-J subscale, leg extension power was also negatively associated with Intrusion (*β* = –0.114, *P* = 0.045) and Hyperarousal (*β* = –0.163, *P* = 0.004) after adjusting for all other significant variables. For women, hypertension (*β*  = 0.226, *P* = 0.032) and depressive symptoms (*β*  = 0.205, *P* = 0.046) were associated with the total score of the IES-R-J.

**Conclusions:**

Leg extension power is a potentially modifiable pre-disaster risk factor among men for attenuating the severity of PTSD symptoms associated with great disasters such as the Great East Japan Earthquake among men.

## Introduction

On 11 March 2011, the Great East Japan Earthquake and Tsunami devastated the northeastern coast of Japan and left approximately 18,500 dead or missing. Further, about 200,000 survivors were forced to live in uncomfortable environments after they were evacuated [1], [2]. The survivors were not only damaged both physically and mentally, but they were also forced to change their lifestyle habits during the peri-disaster period. Post-traumatic stress disorder (PTSD) is a common psychological problem following natural disasters, including earthquakes and tsunamis [3]. PTSD is associated with suicide attempts and high health care costs [4], [5]; therefore, it is important to identify the risk factors for PTSD to enable earlier detection and provide more urgently needed support for those suffering from PTSD after natural disasters.

Previous studies have identified three categories of PTSD risk factors: pre-disaster (e.g., gender, education level, psychiatric history); peri-disaster (e.g., degree of exposure to the event); and post-disaster (e.g., lack of social support) [6], [7]. Compared with peri- and post-disaster risk factors, pre-disaster risk factors are more important from the standpoint of preparing for unexpected large-scale disasters in countries that are prone to them, such as Japan [8]. Such advanced planning is necessary to prevent the declining mental health that naturally follows as a result of these disasters. As mentioned previously, the existing literature on pre-disaster risk factors has been limited to either sociodemographic factors or psychiatric history, neither of which can be changed easily to ensure a more positive outcome for disaster survivors. Detection of modifiable risk factors, such as physical function and lifestyle-related factors, may not only enable the early discovery of a PTSD prone individual, but it also may make it possible to develop PTSD resistance in those who would be prone to it.

Under non-disaster ordinary conditions, low physical function is associated with increased risk of depression [9], depression morbidity [10] or the persistence of depressive and anxiety disorders [11]. These findings suggest the possibility that even under disaster conditions, lower physical function could be associated with poor mental health. Tsai et al. showed that participants with posttraumatic stress symptoms (PTSS) had a lower score in both the physical component summary and the physical functioning subscales of the Medical Outcomes Study Short Form-36 (MOS SF-36) at both six months and three years after the Chi-Chi earthquake [12]. This result indicates that poorer mental health after the earthquake had a significant negative influence on future physical functioning, though physical function level before the earthquake was not evaluated in the previous study. Accordingly, it remains possible that participants with PTSS could have already had a lower physical function level before the earthquake, suggesting the possibility that physical function could be a modifiable risk factor for PTSD after disasters.

In addition, it is well-established that healthy behavior intervention contributes to mental health as well as physical health [13]. Under disaster conditions, there are two opposite possibilities for the associations between lifestyles and PTSD. One is that people with healthy lifestyle could preserve their mental health even under a stressful situation, and the other is that, rather, they could have higher stress level from disrupting their lifestyle, leading to more stressful refuge life. Given that previous studies reported that an individual’s lifestyle after a disaster has also been associated with PTSD among war veterans [14]–[16], lifestyle before disasters also could be associated with PTSD after disasters or life-disrupting, stress-inducing situations. However, the associations of pre-disaster factors with PTSD cannot be examined, unless these factors had been examined in an organized survey before disasters.

We conducted the Oroshisho longitudinal study, which comprised a cohort of adult employees working at the Sendai Oroshisho Center in Sendai, the area close to the epicenter of the earthquake, from August 2008 to August 2011 [17]–[20]. This study aimed to investigate the risk factors for lifestyle-related illness, including metabolic syndrome. In this study, we designed a one-year retrospective cohort study to examine the association between pre-disaster lifestyle, physical functioning, and PTSD symptoms, which was evaluated by a self-report questionnaire administered to survivors of the Great East Japan Earthquake post-disaster.

## Methods

### Study Participants

**Figure 1:**
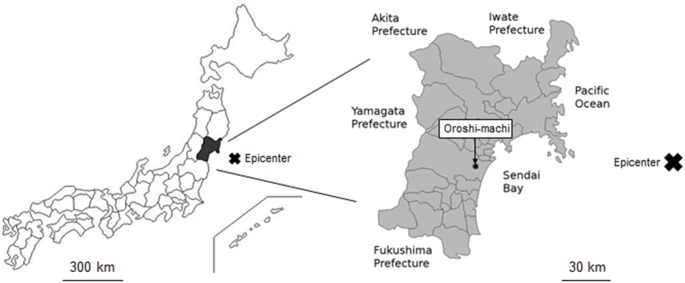
Location of the epicenter of the Great East Japan Earthquake and Oroshi-machi. (a) The epicenter of the East Japan Earthquake (cross) was under the Pacific Ocean about 150 km east of the Miyagi Prefecture. (b) The Sendai Oroshisho Center is located in Oroshi-machi in Eastern Sendai, where the tsunami approached within 2–3 km distance.

The participants were employees of the Sendai Oroshisho Center, a group of over 120 small and medium enterprises in Sendai, Miyagi prefecture, Japan (Figure 1). The Sendai Oroshisho Center is located in Oroshi-machi in Eastern Sendai, where the tsunami approached within 2–3 km distance, and many buildings were damaged or destroyed by the earthquake. Most participants live in the Miyagi prefecture, where the disaster affected normal life for more than one month [21].

**Figure 2:**
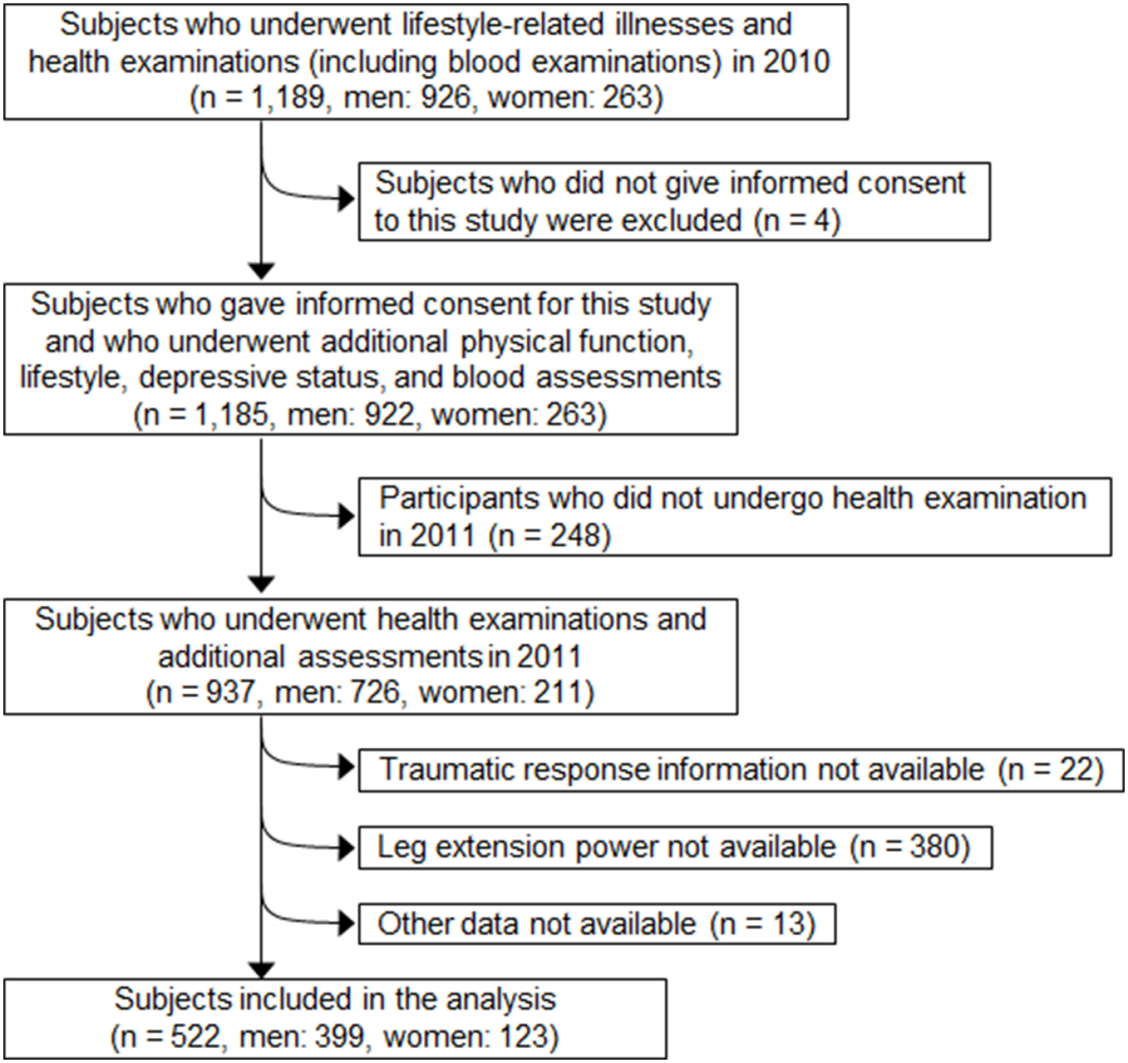
Flow chart of the sample selection process.

The sample selection process was as follows (Figure 2). In 2010, 1189 participants (926 men and 263 women) received annual health examinations for lifestyle-related illnesses, including anthropometric measurements, hematological examinations, and additional assessments of lifestyle and physical function. Of these, 1185 (922 men and 263 women) participated in our survey. Those who did not undergo health examinations in 2011 (n = 248) were excluded. Moreover, we excluded 22 participants who had not answered the Impact of Event Scale-Revised (IES-R) and 380 participants who had not been measured for leg extension power. We also excluded 13 participants due to incomplete data. Final analyses included 522 participants (399 men and 123 women).

### Ethics Statement

The protocol of this study was approved by the Institutional Review Board of the Tohoku University Graduate School of Medicine. After a complete description of the study was provided to the subjects, written informed consent was obtained from each of them.

### PTSD Symptoms

The Impact of Event Scale-Revised (IES-R) [22], a short, easily administered self-report questionnaire, was used to evaluate probable PTSD in August 2011. The IES-R uses 22 items and a four-point Likert-type scale to assess the three most common symptoms of PTSD: intrusion, avoidance, and hyperarousal. It is the most widely used measure internationally in all forms of disaster area research, and the both the validity and reliability of the Japanese version of the IES-R (IES-R-J) has been confirmed [23]. The IES-R-J was useful in identifying survivors with PTSD symptoms as a clinical concern (full and partial PTSD) after various kinds of traumatic events [23]. In this study, the total scale and subscale (Intrusion, Avoidance, and Hyperarousal) scores were calculated. Moreover, we used the cut-off of 24/25 proposed by Asukai [23], as the cut-off was applied to survivors of a disaster, where individuals with a total IES-R-J score greater than or equal to 25 were regarded as having “probable PTSD.” The internal reliability for this study was 0.93.

### Physical Function Factor

In order to evaluate physical function, maximal bilateral leg extension power (W) was determined by using an isotonic apparatus (Anaeropress 3500; Combi Co., Tokyo, Japan). After warming up, the participants reclined on the seat and placed both feet on the footplate at a knee angle of 90°. The load of the footplate was set to the subject’s weight. Subjects pressed their feet horizontally as intensely as possible until their legs extended fully. Five trials were performed at 15-s intervals, and the highest value was chosen for inclusion in the analyses. The reliability and validity of the leg extension power measurement were evaluated and described in details elsewhere [24].

### Lifestyle-related Factors

Information on smoking status (never, former, or current smoker), alcohol-drinking status (never, 1–6 day(s)/week, or 7 days/week), and sleep duration (6–8 hours, <6, or >8 hours) was obtained from a questionnaire survey. Levels of daily physical activity (PA) were estimated using the International Physical Activity Questionnaire (Japanese version) [25], and the responses were divided into three categories (<1, 1–22, or ≥23 metabolic equivalent of tasks (METs) × hours/week). More than 23 METs × hours/week is the quantity of PA and exercise recommended for the promotion of health by the Ministry of Health, Labour and Welfare of Japan [26]. The frequency of breakfast consumption was assessed with the question “How many times do you eat breakfast a week?” and the responses were categorized into two groups: those who ate breakfast≥4 times/week and those who ate breakfast less than four times per week [27]. Frequency of daily toothbrushing in the preceding month was assessed by a self-reported questionnaire, and participants were categorized into two groups: those who brushed ≥3 times/day and those who brushed less than three times/day.

### Physical Condition Factors

Blood pressure (BP) was measured twice in each participant’s left upper arm using an automatic device (YAMASU605P; Kenzmedico Co. Ltd., Saitama, Japan) after a five-minute resting period in a seated position. The mean values of systolic and diastolic BP were used as the BP values, respectively. Blood samples were drawn from the antecubital vein, with minimal tourniquet use, while the subjects were still seated. The specimens were collected in siliconized vacuum glass tubes containing sodium fluoride for fasting blood glucose and no additives for lipid analyses. Fasting blood glucose concentration was measured by enzymatic methods (Eerotec, Tokyo, Japan). The triglyceride, low-density lipoprotein cholesterol, and high-density lipoprotein cholesterol concentrations were measured by enzymatic methods using the appropriate kits (Sekisui Medical, Tokyo, Japan).

Histories of diabetes, hypertension, and dyslipidemia, and current treatments for each were evaluated on the basis of yes or no responses. Participants were considered to have diabetes if they had hyperglycemia (a fasting blood glucose level ≥126 mg/dL) or were receiving treatment for diabetes. Hypertension was identified if a measured systolic blood pressure was ≥140 mmHg or a measured diastolic blood pressure ≥90 mmHg, or if the participant reported being treated for hypertension. Dyslipidemia was identified if a measured blood triglyceride level was ≥150 mg/dL, if a measured blood LDL-cholesterol level was ≥140 mg/dL, if a measured blood HDL-cholesterol level was <40 mg/dL, or if the participant reported being treated for dyslipidemia.

Depressive symptoms were assessed according to the Japanese version of the Self-Rating Depression Scale (SDS) [28]. Participants were defined as depressed when the SDS score was 45 or greater [29].

### Sociodemographic Factors

Sociodemographic variables, including age, educational level (lower than college level or college level and above), occupation (desk-based or not), marital status (married or unmarried), and living arrangements (alone or with others) were also assessed by a self-reported questionnaire.

### Disaster-related Factors

Disaster-related factors were evaluated by the self-reported questionnaire, and participants were categorized into two or three groups based on their responses: family loss (yes or no); property damage (completely destroyed, partially damaged, or other); and work volume (unchanged, increased, or decreased).

### Statistical Analysis

The distributions of all continuous variables in this study were positively skewed; therefore, they were normalized by log-transformation for our analyses. When we calculated log-transformed IES-R-J, 1.0 was added [total IES-R score+1] before transformation. Descriptive data were expressed as the median (interquartile range) for continuous variables and as percentages for categorical variables. To identify factors associated with PTSD symptoms, linear regression analysis was performed. Log-transformed total and subscale scores of IES-R-J in 2011 were used as dependent variables, and all other variables assessed in 2010 and disaster-related factors assessed in 2011 were used as independent variables. Multivariate linear regression was used to calculate slope (B), standard error (SE), and *β*, after controlling simultaneously for all variables, including: leg extension power (log-transformed); lifestyle-related factors: PA (<1 METs·hours/week, 1–22 METs·hours/week, or ≥23 METs·hours/week); smoking status (never, former, or current); drinking status (never, 1–6 day(s)/week, or 7 days/week); sleep duration (6–8 hours/day or not); tooth brushing (≥3 times/day or <3 times/day); and eating breakfast (<4 times/week or ≥4 times/week). Additional physical condition factors were also assessed, including: diabetes (no or yes), hypertension (no or yes), dyslipidemia (no or yes), and depressive symptoms (SDS≥45). Sociodemographic factors were also included in the analysis in the following form: age (log-transformed), education (≥ college or < college), occupation (deskwork or non-desk work), marital status (unmarried or married), and living alone (no or yes). The disaster-related factors were family loss (no or yes); property damage (other, partially damaged, or completely damaged); and work volume (unchanged, increased, or decreased). Multicollinearity was assessed by using the variance inflation factor (VIF) [30]. A VIF exceeding 10 is regarded as indicating serious multicollinearity, and values greater than 4.0 may be a cause for concern [30].

All tests for statistical significance were two-sided, and *P*<0.05 was defined as being the marker of statistical significance. All statistical analyses were performed using SPSS 17.0 for Windows (SPSS, Inc., Chicago, IL, USA).

## Results

Tables 1 and 2 show the baseline characteristics for men and women, respectively. Among men, the median (interquartile range) and range of the total score of the IES-R-J among men were 10.0 (3.0–19.0) and 0.0–77.0, respectively, and the prevalence of probable PTSD (IES-R-J≥25) was 14.3%. For women, the median (interquartile range) and range of the total score of the IES-R-J were 16.0 (7.0–24.0) and 0.0–51.0, respectively, and the prevalence of probable PTSD was 24.4%. The ages of the participants ranged from 22 to 84 and 22 to 81 for men and women, respectively.

**Table 1:** Participants characteristics and pre-disaster factors associated with the total score of IES-R-J in men (n = 399)^a^.

	Value^b^	Bivariate analysis				Multivariate analysis			
		B	SE	*β*	*P*	B	SE	*β*	*P*
IES-R-J									
Total^c^	10.0 (3.0–19.0)								
Intrusion^c^	4.0 (1.0–7.0)								
Avoidance^c^	4.0 (1.0–8.0)								
Hyperarousal^c^	3.0 (1.0–5.0)								
IES-R-J≥25, %	14.3								
*Sociodemographic factors*									
Age (years)^c^	45.0 (37.0–55.0)	0.173	0.219	0.040	0.430	–0.310	0.279	–0.071	0.268
Education									
<college, %	60.4	Ref.				Ref.			
≥college, %	39.6	**–0.106**	**0.047**	**–0.114**	**0.023**	–0.088	0.049	–0.094	0.070
Occupation									
Desk work, %	51.6	Ref.				Ref.			
Non-desk work, %	48.4	0.075	0.046	0.082	0.101	0.071	0.046	0.078	0.125
Marital status									
Unmarried, %	23.1	Ref.				Ref.			
Married, %	76.9	0.010	0.054	0.009	0.853	0.075	0.072	0.069	0.294
Living alone									
No, %	89.0	Ref.				Ref.			
Yes, %	11.0	0.009	0.073	0.006	0.897	0.023	0.092	0.016	0.803
*Physical function factor*									
Leg extension power (W/kg)^c^	18.5 (15.0–21.8)	**–0.448**	**0.171**	**–0.130**	**0.009**	**–0.438**	**0.195**	**–0.128**	**0.025**
*Lifestyle factors*									
PA									
<1 METs·hours/week, %	23.8	Ref.				Ref.			
1–22 METs·hours/week, %	36.3	**–0.105**	**0.047**	**–0.111**	**0.027**	–0.075	0.062	–0.078	0.227
≥23 METs·hours/week, %	39.9	0.070	0.047	0.075	0.136	0.011	0.061	0.012	0.856
Smoking status									
Never, %	42.9	Ref.				Ref.			
Former, %	14.5	–0.091	0.065	–0.070	0.162	–0.094	0.071	–0.073	0.182
Current, %	42.6	0.071	0.046	0.077	0.124	0.006	0.052	0.007	0.901
Drinking status									
Never, %	17.3	Ref.				Ref.			
1–6 day(s)/week, %	51.1	–0.507	0.046	–0.062	0.217	0.129	0.066	0.141	0.052
7 days/week, %	31.6	**0.135**	**0.049**	**0.138**	**0.006**	**0.200**	**0.072**	**0.203**	**0.006**
Sleep duration									
6–8 hours/day, %	56.9	Ref.				Ref.			
<6 or >8 hours/day, %	43.1	–0.052	0.046	–0.056	0.265	–0.033	0.049	–0.036	0.494
Tooth brushing									
≥3 times/day, %	88.5	Ref.				Ref.			
<3 time/day, %	11.5	–0.063	0.072	–0.044	0.380	–0.024	0.074	–0.017	0.747
Eating breakfast									
<4 times/week, %	34.1	Ref.				Ref.			
≥4 times/week, %	65.9	0.025	0.048	0.026	0.607	0.040	0.051	0.041	0.431
*Physical condition factor*									
Diabetes									
No, %	93.0	Ref.				Ref.			
Yes, %	7.0	0.111	0.090	0.062	0.214	0.049	0.094	0.027	0.52
Hypertension									
No, %	70.7	Ref.				Ref.			
Yes, %	29.3	0.045	0.050	0.045	0.371	0.009	0.056	0.008	0.880
Dyslipidemia									
No, %	53.6	Ref.				Ref.			
Yes, %	46.4	0.007	0.046	0.007	0.885	0.004	0.047	0.004	0.930
Depressive symptoms									
SDS<45, %	72.4	Ref.				Ref.			
SDS≥45, %	27.6	**0.135**	**0.051**	**0.132**	**0.008**	**0.142**	**0.053**	**0.139**	**0.008**
*Disaster-related factors* d									
Family loss									
No, %	98.7	Ref.				Ref.			
Yes, %	1.3	0.316	0.205	0.077	0.125	0.272	0.223	0.066	0.223
Property damage									
Other, %	57.2	Ref.				Ref.			
Partially-damaged, %	37.3	0.030	0.047	0.032	0.525	0.033	0.048	0.035	0.496
Completely-destroyed, %	5.5	0.165	0.100	0.083	0.100	0.092	0.111	0.046	0.404
Work volume									
Unchanged, %	54.1	Ref.				Ref.			
Increased, %	35.6	0.052	0.048	0.055	0.275	0.047	0.050	0.049	0.349
Decreased, %	10.3	0.018	0.075	0.012	0.816	0.033	0.078	0.022	0.677

a IES-R-J, the Japanese version of the Impact of Event Scale-Revised; PA, physical activity; METs, metabolic equivalent of tasks; SDS, Self-rating Depression Scale.

b Data are summarized by median (interquartile range) for continuous variables and by percentage for category variables.

c All continuous variables have been log-transformed.

d Data was measured in 2011 only.

**Table 2:** Participants characteristics and pre-disaster factors associated with the total score of IES-R-J in women (n = 123)^a^.

	Value^b^	Bivariate analysis				Multivariate analysis			
		B	SE	*β*	*P*	B	SE	*β*	*P*
IES-R-J									
Total^c^	16.0 (7.0–24.0)								
Intrusion^c^	5.0 (2.0–9.0)								
Avoidance^c^	5.0 (3.0–10.0)								
Hyperarousal^c^	4.0 (2.0–7.0)								
IES-R-J≥25, %	24.4								
*Sociodemographic factors*									
Age (years)^c^	38.0 (35.0–50.0)	–0.141	0.311	–0.041	0.650	–0.720	0.449	–0.207	0.112
Education									
<college, %	87.8	Ref.				Ref.			
≥college, %	12.2	0.000	0.105	0.000	0.998	–0.065	0.115	–0.056	0.573
Occupation									
Desk work, %	85.4	Ref.				Ref.			
Non-desk work, %	14.6	0.014	0.098	0.013	0.884	0.009	0.111	0.008	0.937
Marital status									
Unmarried, %	50.4	Ref.				Ref.			
Married, %	49.6	–0.027	0.069	–0.035	0.698	0.052	0.095	0.068	0.583
Living alone									
No, %	87.0	Ref.				Ref.			
Yes, %	13.0	0.001	0.103	0.001	0.995	0.041	0.125	0.035	0.744
*Physical function factor*									
Leg extension power (W/kg)^c^	10.2 (7.9–13.2)	0.093	0.201	0.042	0.646	0.285	0.229	0.130	0.216
*Lifestyle factors*									
PA									
<1 METs·hours/week, %	30.1	Ref.				Ref.			
1–22 METs·hours/week, %	45.5	0.012	0.069	0.016	0.857	–0.011	0.092	–0.014	0.905
≥23 METs·hours/week, %	24.4	–0.056	0.080	–0.064	0.485	0.080	0.112	0.090	0.479
Smoking status									
Never, %	72.4	Ref.				Ref.			
Former, %	6.5	–0.118	0.139	–0.077	0.400	–0.203	0.161	–0.132	0.212
Current, %	21.1	0.042	0.084	0.045	0.622	0.038	0.097	0.040	0.700
Drinking status									
Never, %	41.5	Ref.				Ref.			
1–6 day(s)/week, %	50.4	–0.098	0.068	–0.129	0.156	–0.091	0.083	–0.120	0.274
7 days/week, %	8.1	0.115	0.126	0.083	0.362	0.155	0.147	0.112	0.291
Sleep duration									
6–8 hours/day, %	52.8	Ref.				Ref.			
<6 or >8 hours/day, %	47.2	0.015	0.069	0.020	0.827	0.004	0.079	0.005	0.959
Tooth brushing									
≥3 times/day, %	71.5	Ref.				Ref.			
<3 time/day, %	28.5	0.019	0.077	0.022	0.808	0.061	0.084	0.073	0.470
Eating breakfast									
<4 times/week, %	38.2	Ref.				Ref.			
≥4 times/week, %	61.8	0.029	0.071	0.037	0.688	0.039	0.084	0.049	0.646
*Physical condition factor*									
Diabetes									
No, %	98.4	Ref.				Ref.			
Yes, %	1.6	0.084	0.273	0.028	0.757	0.138	0.298	0.046	0.644
Hypertension									
No, %	91.9	Ref.				Ref.			
Yes, %	8.1	**0.279**	**0.124**	**0.201**	**0.026**	**0.329**	**0.151**	**0.226**	**0.032**
Dyslipidemia									
No, %	77.2	Ref.				Ref.			
Yes, %	22.8	–0.020	0.082	–0.023	0.804	–0.026	0.100	–0.028	0.799
Depressive symptoms									
SDS<45, %	65.9	Ref.				Ref.			
SDS≥45, %	34.1	**0.169**	**0.071**	**0.212**	**0.019**	**0.164**	**0.081**	**0.205**	**0.046**
*Disaster-related factors* d									
Family loss									
No, %	98.4	Ref.				Ref.			
Yes, %	1.6	**0.545**	**0.268**	**0.182**	**0.044**	0.194	0.444	0.065	0.664
Property damage									
Other, %	50.4	Ref.				Ref.			
Partially-damaged, %	46.3	–0.030	0.069	–0.039	0.665	–0.009	0.079	–0.012	0.909
Completely-destroyed, %	3.3	0.352	0.192	0.165	0.069	0.154	0.306	0.072	0.615
Work volume									
Unchanged, %	60.2	Ref.				Ref.			
Increased, %	32.5	**0.170**	**0.072**	**0.210**	**0.020**	0.160	0.087	0.196	0.068
Decreased, %	7.3	–0.004	0.132	–0.002	0.978	0.079	0.147	0.054	0.591

a IES-R-J, the Japanese version of the Impact of Event Scale-Revised; PA, physical activity; METs, metabolic equivalent of tasks; SDS, Self-rating Depression Scale.

b Data are summarized by median (interquartile range) for continuous variables and by percentage for category variables.

c All continuous variables have been log-transformed.

d Data was measured in 2011 only.

Table 1 also shows the pre-disaster factors associated with the total score of IES-R-J among men five months after the earthquake. In bivariate linear regression analysis, the total score of IES-R-J was negatively associated with having a college education (*β* = –0.114, *P* = 0.023), leg extension power (*β* = –0.130, *P* = 0.009), and engagement in 1–22 METs·hours/week of physical activity (<1 METs·hours/week was used as the reference, *β* = –0.111, *P* = 0.027). Moreover, daily drinking (no drinking was used as the reference, *β*  = 0.138, *P* = 0.006) and depressive symptoms (*β*  = 0.132, *P* = 0.008) were positively associated with the total score of IES-R-J.

To identify the pre-disaster risk factors associated with PTSD symptoms among men, multivariate linear regression analysis was performed using all variables listed in Table 1 as independent variables. Leg extension power (*β* = –0.128, *P* = 0.025), daily drinking (*β*  = 0.203, *P* = 0.006), and depressive symptoms (*β*  = 0.139, *P* = 0.008) were associated with total IES-R-J scores. Moreover, for the IES-R-J subscales, leg extension power was negatively associated with Intrusion (*β* = –0.114, *P* = 0.045) and Hyperarousal (*β* = –0.163, *P* = 0.004) after adjusting for the above-mentioned variables (Table 3).

**Table 3:** Relationship of leg extension power with each subscale scores of IES-R-J among men and women^a^.

	Men (n = 399)					Women (n = 123)			
	B	SE	*β*	*P*		B	SE	*β*	*P*
IES-R-J^b^									
Intrusion	–0.327	0.162	–0.114	0.045		0.200	0.215	0.099	0.355
Avoidance	–0.233	0.154	–0.086	0.131		0.159	0.199	0.085	0.428
Hyperarousal	–0.425	0.148	–0.163	0.004		0.134	0.192	0.071	0.486

a Adjusted for physical activiy (<1 METs·hours/week, 1–22 METs·hours/week, or≥23 METs·hours/week), smoking status (never, former, or current), drinking status (never, 1–6 day(s)/week, or 7 days/week), sleep duration (6–8 hours/day or not), tooth brushing (≥3 times/day or <3 times/day), eating breakfast (<4 times/week or ≥4 times/week), diabetes (no or yes), hypertension (no or yes), dyslipidemia (no or yes), depressive symptoms (SDS≥45), age (log-transformed), education (≥ college or < college), occupation (deskwork or non-desk work), marital status (unmarried or married), family loss (no or yes), property damage (other, partially damaged, or completely damaged), and work volume (unchanged, increased, or decreased).

b All continuous variables have been log-transformed.

On the other hand, among women (Table 2), hypertension (*β*  = 0.201, *P* = 0.026) and depressive symptoms (*β*  = 0.212, *P* = 0.019) were positively associated with total IES-R-J scores. In addition, family loss (*β*  = 0.182, *P* = 0.044) and increased work volume after the earthquake (unchanged work volume was used as the reference, *β*  = 0.210, *P* = 0.020) were associated with total IES-R-J scores. The associations of the total IES-R-J scores with hypertension (*β*  = 0.226, *P* = 0.032) and depressive symptoms (*β*  = 0.205, *P* = 0.046) remained significant even after adjusting for all variables listed in Table 2.

Evidence for multicollinearity was absent because the variance inflation factor for independent variables for all models in Tables 1 and 2 was less than 2.0.

## Discussion

Using a one-year retrospective cohort design, this study examined the relationship between the prevalence of probable PTSD and modifiable pre-disaster factors, such as lifestyle or physical functioning. We found that, among men, lower leg extension power and daily drinking before the earthquake were associated with higher total IES-R-J scores, even after adjusting for disaster-related factors. In addition, among women, participants with hypertension before the earthquake also had higher total IES-R-J scores. Although this study has several limitations, as described below, we were able to demonstrate that pre-disaster physical functioning and condition, which were free from the direct influence of the disaster, are risk factors for probable PTSD post-disaster, owing to the evidence from the pre-existing cohort study.

Previous research examining the associations between physical functioning and mental health following disasters has been limited [12]. Tsai et al. reported that people with PTSS symptoms six months and three years after the Chi-Chi earthquake in Taiwan had poorer self-reported physical functioning than controls, and their physical component summary scores on the MOS SF-36 negatively correlated with their PTSS symptoms three years post-disaster [12]. These findings indicated that poorer mental health after the disaster had a significant negative influence on future physical functioning. However, in our study, lower pre-disaster leg extension power was associated with the severity of PTSD symptoms following the Great East Japan Earthquake. Consistent with the associations between poorer physical functioning and a higher risk of poor mental health under non-disaster conditions [10], [11], [31], higher pre-disaster physical functioning may have a protective influence on disaster-related probable PTSD; thus, daily maintenance and enhancement of physical functioning may primarily prevent disaster-related PTSD.

Our findings might be partly explained by behavioral mechanisms. Poorer physical functioning has been associated with fewer friendship contacts, fewer family contacts, and less perceived peer support [32]. Lower walking speed has also been associated with lower social participation as defined by taking part in social, cultural, and leisure activities [33]. Taken together, it may be the case that survivors of natural disasters with less leg extension power are sensitive to stress, as they have fewer opportunities to participate in social activities or less contact with others under disaster conditions. Thus, leg extension power might not be the sole influencing factor.

In addition to the effect of social contact, exercise per se can help people to cope with stress and other problem for life event [34], [35], and exercise is recommended as a stress management technique [34]. A previous study reported that physical fitness, which is strongly related to exercise, was associated with decreased psychological distress among community-dwelling adults, and the stressor–distress relationship was moderated by physical fitness [36]. Similar association was confirmed in Gerber’s study which recruited employees of police force and emergency response service corps [37]. Although mechanisms for the association are still unknown, these results suggested that physical function could buffer against the effects of real-life stress [38]. Therefore, even under disaster condition, higher physical function may be expected to have a protective effect against disaster-induced stress.

One of the strengths of our study was that the leg extension power measurement was performed before the disaster. As compared with physical performance, self-perceived measures are likely to be affected by mental conditions such as depressive symptoms, resulting in lower objectivity. Therefore, the self-reported questionnaire is more susceptible to information bias, and the influence of this bias on self-reported measures under disaster conditions can be greater than under non-disaster conditions. In addition, even a performance-based measure, like the level of physical functioning measured following a disaster could be underestimated when compared to the actual level of leg extension power. Thus, because our study used a pre-disaster measure of leg extension power, our results were less susceptible to information bias and had higher internal validity, as compared with previous studies [12].

Previous studies have reported that PTSD was associated with cardiometabolic diseases [39], [40]. In a previous cross-sectional study, Australian veterans with a history of PTSD had an increased odds ratio of having hypertension [41]. Interestingly, our study demonstrated that among women, those with hypertension before the earthquake had a higher total score of IES-R-J after the disaster. In the Great East Japan Earthquake, people, including our study participants, experienced great difficulties in obtaining food and beverages, and many were without medication for more than one month, due to significant damages of the public transportation system and fuel shortage [21], [42]. The relief food supplies provided in the first couple of weeks were mainly unhealthy foods and those high in carbohydrates and sodium [43], and it is very likely that people with hypertension had greater trouble controlling their diets or taking medications, because treatment of hypertension has been correlated with improvement in mood and quality of life [44]. Thus, this finding provides important clinical data regarding the need to take care of survivors being treated for hypertension when a natural disaster occurs.

Trauma exposure by natural disasters was positively associated with alcohol consumption [45], even after adjusting for pre-disaster alcohol consumption in previous studies [46]. Interestingly, in this study, pre-disaster daily drinking had a positive association with PTSD symptom severity among adult men. It was found that, after the Great Hansin Earthquake on January 17, 1995, in Japan, the quantity of alcoholic beverages sold in a disaster-stricken prefecture after the earthquake decreased after adjusting for population movement and the decreased number of retail shops after the disaster [47]. One possible reason for this decrease in alcohol consumption is assumed to be the strong self-discipline of Japanese people and a culture that respects such behavior [47]. Given that stress coping is one of the motives for drinking alcohol, which also predicts the amount of alcohol consumption [48], our findings suggest that those who drank daily before the disaster had to reduce their drinking after the earthquake, which might have rendered them susceptible to stress, thereby exacerbating the severity of PTSD symptoms.

This study had several limitations. First, PTSD was not assessed by an interview; however, the IES-R seems to be a reasonable assessment method for screening probable PTSD in emergency situations such as disasters. Second, we could not exclude the influence of other factors affecting PTSD. For example, prior history of psychiatric disease is an important risk factor for developing PTSD [6], but it is difficult to precisely evaluate mental health problems in this setting, because previous reviews reported that Japanese people tend to keep a greater social distance from individuals with mental illness. Stigmatizing attitudes toward mental health patients in Japan are stronger than they are in other Asian countries [49]. Third, although we designed a retrospective cohort study, we could not exclude participants who already had PTSD in 2010. This is because the Oroshisho study was conducted in 2008 to examine risk factors for lifestyle-related illnesses, and it did not evaluate PTSD symptoms before 2011. However, we excluded those who had depressive symptoms, which is a strong risk factor for PTSD [50]. Furthermore, given that the 12-month prevalence of PTSD among Japanese adults is 0.4% [51], the influence of previous PTSD history could not have been substantial. Finally, the sample size was small, particularly lacking in women respondents, and unemployed people were not included. The generalizability of our findings to all survivors of the Great East Japan Earthquake is limited because our participants were relatively moderately harmed by the earthquake. However, given that PTSD is a common mental health problem, it is important to identify risk factors for PTSD to enable earlier detection and provide more urgent support among not only those who are severely affected with the disorder, but also those for whom PTSD even moderately affects their daily functioning.

In conclusion, the relationships between the severity of PTSD symptoms and pre-disaster factors were examined using data obtained before the disaster. The results showed that, among men, lower leg extension power and daily drinking before the earthquake were associated with higher PTSD symptom severity five months after the Great East Japan Earthquake. In addition, among women, participants with hypertension before the earthquake also had higher total IES-R-J scores. Thus, daily maintenance and improvement of physical function could be one of the primary ways to prevent declining mental health following disaster situations.
